# Retrospective Analysis of Suspensory Ligament Branch Injuries in 70 Dressage Horses

**DOI:** 10.3390/ani15213079

**Published:** 2025-10-23

**Authors:** Ana Boado, Danica Pollard, Sue Dyson

**Affiliations:** 1 Independent Researcher, Avenida Salmoral 4, Manzanares el Real, 28492 Madrid, Spain; 2 Medicine and Surgery Department, Universidad Complutense de Madrid, Av. Complutense, Moncloa-Aravaca, 28040 Madrid, Spain; 3 Independent Researcher, Rodham Road, Christchurch, Wisbech PE14 9NU, Cambridgeshire, UK; drdee.pollard@gmail.com; 4 Independent Researcher, Church Road, Market Weston, Diss IP22 2NX, Suffolk, UK; sue.dyson@aol.com

**Keywords:** desmitis, fetlock, lameness, ultrasonography, power Doppler, osteoarthritis, radial pressure wave therapy, periligamentous fibrosis

## Abstract

There are no studies that have investigated factors influencing the outcome of dressage horses with suspensory ligament (SL) branch injuries. The aim was to determine if age, breed, work level, injury severity, anatomical localisation of injury, number of injured branches, periligamentous fibrosis, persistence of power Doppler signal or coexistent osteoarthritis of a metacarpophalangeal (MCP) or metatarsophalangeal (MTP) joint influenced the prognosis of 70 dressage horses. Outcome was defined as good (return to pre-injury level of work or higher), poor (return to a lower level of work) or retirement. Chi-squared or Fisher’s exact test and the Kruskal–Wallis test were used to identify relationships between variables of interest and follow-up outcome. Follow-up outcome was good in 44/70 horses (62.9%), poor in 13/70 (18.6.%) and 13/70 horses (19.1%) were retired due to no response to treatment. Ultrasonographic lesion grade (*p* = 0.07), cross-sectional area (CSA) of the SL (*p* = 0.96), CSA of the lesion (*p* = 0.28) and the lesion CSA as a percentage of the SL CSA (*p* = 0.40) were not associated with outcome. Power Doppler signal was present in 75.8% of injured branches at the initial examination. The severity of power Doppler signal was not associated with outcome (*p* = 0.20); however, persistence of power Doppler signal was negatively associated with outcome (*p* < 0.001). Other variables did not influence the follow-up outcome. Early recognition of SL branch injury is likely to result in a more favourable outcome with appropriate treatment and management.

## Simple Summary

There are limited studies investigating the outcome for suspensory ligament (SL) branch injuries in sports horses, and none that focus on dressage horses. The aim was to describe the clinical and ultrasonographic features of SL branch injuries in 70 dressage horses and the response to treatment. There were 43 Warmbloods, 22 Iberian and 5 cross-breed horses, with a median age of 9 years. Geldings (64.3%) and stallions (31.4%) predominated. There were 74 limbs affected (59.5% forelimbs and 41.9% hindlimbs) and 89 branches; lateral branch injuries (75.3%) exceeded medial branch injuries (27.0%). Pain was elicited on firm palpation of 84.3% of injured branches. The most frequent lameness grade was 2/5. Most (66.3%) injuries were localised to the distal one-third of the branch. Ultrasonographic grades were mild (22.5%), moderate (48.3%) and severe (29.2%). In total, 63 percent of horses returned to the same level of work as pre-injury or higher, of which 31.3% were treated conservatively by modification of the exercise programme and serial ultrasonographic monitoring; 76.2% of these horses also received radial pressure wave therapy. Horses with severe injuries were more likely to be retired than those with mild or moderate injuries. Early diagnosis is most likely to result in a successful outcome.

## 1. Introduction

The third interosseous ligament or suspensory ligament (SL) can be divided into three regions of clinical relevance: the origin, the body and the branches [1]. Each branch inserts into the abaxial aspect of the proximal sesamoid bones (PSBs) and approximately the distal one-third of both branches are sub-synovial relative to the palmar/plantar pouches of the metacarpophalangeal (MCP) and metatarsophalangeal (MTP) joints [2]. The insertion of these branches is an area of transition between tissues of different properties, the ligament, the enthesis and compact bone, making this area potentially particularly susceptible to injury [3,4]. During maximal extension of the MCP or MTP joints, the PSBs move distally and dorsally and the SL branches act as an articular surface [5]. The SL is under maximum load when the MCP or MTP joint is in extension and relaxes during limb protraction [6].

Suspensory branch desmitis is a common injury in horses [1,7,8,9,10,11,12,13]. Injuries can occur subclinically in Thoroughbred racehorses [9,10,11] and showjumpers [12] or be associated with lameness in horses from a variety of work disciplines [1,13]. Early recognition of suspensory branch desmitis and any concurrent injuries is important to minimise time off work, adopt appropriate treatment and management practices and to mitigate economic losses.

Dressage is an equestrian sport in which a horse is required to perform a series of exercises in an arena with the purpose of demonstrating its natural athletic ability and willingness to perform [14]. Training is essentially repetitive and ideally there is a gradual adaptation of the musculoskeletal system to the loads applied. The aim is for the horse–rider combination to perform the exercises in harmony, with barely perceptible cues, and an absence of signs of discomfort [15]. At upper levels of dressage (Prix St Georges, Intermediate I and II and Grand Prix), the patterns of movements and the biomechanical loads on the musculoskeletal system differ from other equestrian disciplines [16]. The MCP and MTP joints are loaded in extension in some dressage movements such as passage and extended trot [6,17]. Additionally, during many lateral (sideways) movements (for example, half-pass), the SL is subjected to asymmetric loading.

It was previously suggested that dressage horses had a propensity for hindlimb suspensory ligament injuries compared with horses from other equestrian disciplines [18]. In a more recent large-scale study [19] that investigated multifocal suspensory injuries, work discipline was not a risk factor. Despite the increase in interest in dressage, there is limited published information about dressage horse injuries [20].

The objectives of the current study were to describe the clinical features of horses with SL branch injuries, compare the prevalence of medial and lateral suspensory branch desmitis in lame horses, to describe the ultrasonographic findings, to determine the prevalence of concurrent injuries and to establish factors that influence the prognosis for return to full athletic function.

It was hypothesised that 1. the prevalence of lateral suspensory branch desmitis would be higher than medial branch injuries; 2. distal lesions involving the enthesis at the PSB would be more common than more proximal lesions; 3. horses with abaxial lesions would have a better outcome than horses with other anatomical sites of lesions; and 4. horses with severe injuries would have a poorer outcome than horses with mild or moderate injuries.

## 2. Materials and Methods

### 2.1. Data Acquisition

Medical records of horses that were in training for or competing in dressage, between 2010 and 2024, and which had clinical signs and ultrasonographic abnormalities consistent with SL branch injury were reviewed. These included horses described in previous publications that fulfilled the inclusion criteria [21,22]. An SL branch injury was considered clinically relevant if there were localised signs of inflammation (heat, oedema, swelling and pain) at the site, or localisation of pain to the fetlock region by local anaesthesia (palmar [proximal to the digital flexor tendon sheath] and palmar metacarpal [distal to the distal aspect of the second and fourth metacarpal/metatarsal bones] nerves, a ‘low-4-point block’), and imaging findings compatible with desmitis. All horses included in the study were examined by one clinician (AB, Diplomate of the American and European Colleges of Veterinary Sports Medicine and Rehabilitation).

Age (years), breed (Warmblood, Iberian or other), sex (mare, gelding, stallion), lameness grade (0–5) (0 = non-lame, 1 = subtle, 2 = mild, 3 = moderate, 4 = severe, 5 = non-weightbearing) and limb were recorded. The level of training or competition when the horse was first examined was classified as 1. a young horse (a horse up to 7 years of age) or amateur (a rider and horse working below Prix St Georges), 2. Prix St Georges, 3. Intermediate I and II and International classes for riders under 25 years of age (U25) and 4. Grand Prix.

Desmitis was classified as a primary suspensory branch injury in the absence of pre-existing osteoarthritis of the MCP or MTP joint. If a horse had pre-existing symptomatic osteoarthritis and subsequently developed suspensory branch desmitis, this was defined as a secondary associated injury.

### 2.2. Clinical Examination

All limbs were palpated systematically in weightbearing and non-weightbearing positions. In a weightbearing position, the uniformity of dorsopalmar (plantar) width of each SL branch was determined [1] and classified as no palpable enlargement, mildly enlarged (just detectable by palpation) or severely enlarged (obvious on visual inspection and palpation). Pressure was applied to each SL branch from proximally to distally with the limb semi-flexed to determine the presence of pain. The presence of effusion in each MCP and MTP joint was recorded and the reaction to passive flexion, documented as no pain or pain on passive flexion. If localised signs of inflammation were obvious, a dynamic examination was not performed to prevent any deterioration of the injury and ultrasonographic examination was performed directly. All other horses were evaluated moving in hand at walk and trot on a firm level surface. The response to distal limb flexion for each limb was recorded (negative, mild, moderate or severe). Perineural anaesthesia was performed systematically [23] in horses that had no localising clinical signs, those in which multifocal pain was suspected based on the clinical examination or those with multilimbed lameness.

### 2.3. Diagnostic Imaging

#### 2.3.1. B-Mode Ultrasonography

The skin was prepared by washing with chlorhexidine scrub solution before application of an alcohol gel; most horses were already clipped, and additional clipping was usually not permitted by the owners or trainers. Ultrasonographic examination of the entire metacarpal and metatarsal regions was performed with the limbs in both weightbearing and non-weightbearing positions, starting proximally from a palmar or plantaro-medial approach.

A 10–14 MHz linear transducer was used (A Z One, Zonare Medical Systems, Mountain View, CA, USA). The SL branches were examined from palmaro(plantaro)lateral and palmaro(plantaro)medial approaches and both transverse and longitudinal images were obtained. A stand-off pad was not used. At the insertion on the PSB, multiple longitudinal images were acquired by moving the transducer from dorsal to palmar or plantar.

#### 2.3.2. Power Doppler Examination

**Figure 1:**
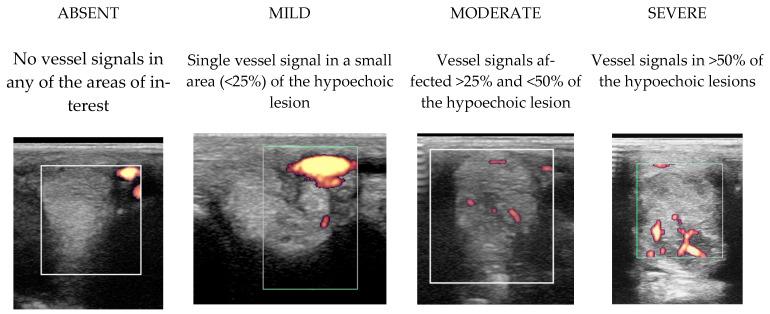
Transverse power Doppler ultrasonographic images of suspensory ligament branches representing the grades of power Doppler signal, from absent to severe, adapted from Szkudlarek et al. [24]. In all images, palmar is to the right.

Power Doppler examination was included for horses examined from 2014 onwards to monitor vascular activity. This was initially performed in a standing position, to assess hypoechoic regions compatible with blood vessels, although if no signal was detected, the examination was repeated in a non-weightbearing position. Hypoechoic regions were assessed in several orientations and for at least two minutes and with the minimal pressure required to achieve adequate image quality, but to avoid false negative results by excessive compression. No sedation was used for examinations to avoid interference with the detection of neovascularisation. Only vascularisation inside a branch was considered a positive finding. If a positive signal was detected, a video clip of several seconds with variable angles was recorded. Power Doppler signal was classified as absent, mild, moderate or severe (Figure 1).

#### 2.3.3. Radiography

Radiographic examination of both metacarpophalangeal and metatarsophalangeal joints was performed using lateromedial, dorsal 15° proximal-palmaro(plantaro)distal oblique, dorsal 45° lateral-palmaro(plantaro)medial oblique and dorsal 45° medial-palmaro(plantaro) lateral oblique views [25]. A portable digital radiography machine was used (Sound Eklin Tru DR LX, Grand Rapids, MI, USA, followed by Varex DR, XR PAD 2, Varex Imaging, Pioneer Road, Salt Lake City, UT, USA (HR 100 µM pixel from 2019 onwards).

### 2.4. Image Interpretation

Images were analysed using open-source DICOM software Horos (version 3.3.6) and measurements were acquired from DICOM images.

#### 2.4.1. Classification of Ultrasonographic Lesions

**Figure 2:**
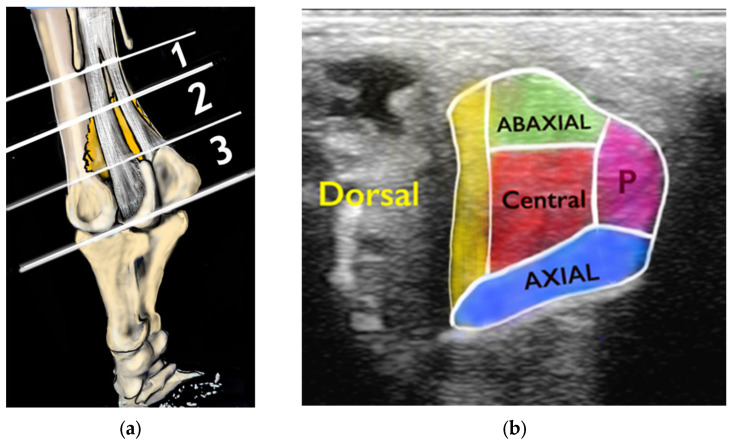
(**a**). Diagram representing the zones into which each suspensory ligament branch was divided from proximal to distal (1 = proximal, 2 = mid, 3 = distal). (**b**). The anatomical location of lesions in a transverse ultrasonographic image. P = palmar.

Ultrasonographic lesions were classified by proximodistal location [11] into three zones: proximal, mid or distal (Figure 2a). The region of abnormality determined from transverse images was defined as axial, abaxial, central, dorsal and palmar or plantar, as previously reported [9] (Figure 2b). An additional lesion category (diffuse) was added when the whole cross-sectional area (CSA) of a branch was affected.

**Figure 3:**
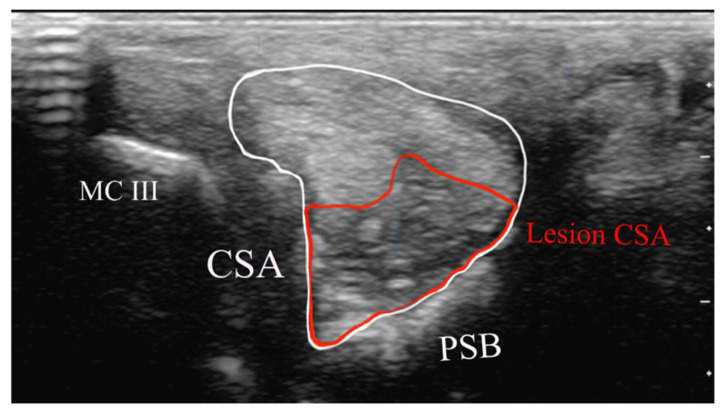
Measurements acquired from the transverse image at the maximal injury zone: cross-sectional area (CSA) of the suspensory branch (white), lesion CSA (red). PSB = proximal sesamoid bone. McIII = third metacarpal bone. There is a thin layer of echogenic tissue subcutaneously.

Measurements were performed by AB at the maximum injury level site from transverse images (Figure 3):

1. Cross-sectional area of the SL branch (cm^2^);
2. Lesion CSA (cm^2^).

Each measurement was repeated five times, and the mean value was calculated. The lesion CSA as a proportion of the total CSA was calculated and expressed as a percentage.

The presence or absence of a subcutaneous hypoechoic area consistent with oedema or echogenic tissue, either uniformly echogenic or heterogeneous in echogenicity, consistent with fibrosis, was recorded.

**Figure 4:**
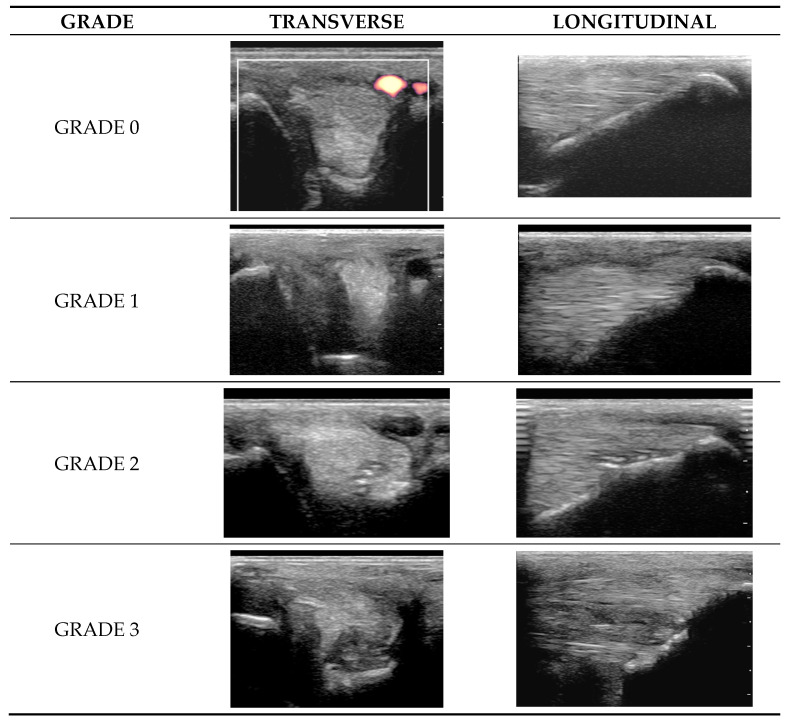
Diagram representing the grades (0–3) detected in both transverse (**left**) and longitudinal (**right**) images. In the transverse images, dorsal is to the left; in the longitudinal images, distal is to the right. Grade 0: homogenous echogenicity, normal size and normal vasculature on the abaxial digital vessel (power Doppler image); normal linear parallel echoes and regular smooth surface of the proximal sesamoid bone (PSB). Grade 1: hypoechogenicity of the abaxial margin of the branch; hypoechogenicity of the superficial fibres and loss of long linear parallel echoes, abaxially close to the enthesis. Grade 2: a focal hypoechoic region <50% of the cross-sectional area (CSA) and with central hyperechoic regions with no associated acoustic shadowing. Image to the right: an area of hypoechogenicity, within which are focal hyperechoic lesions and irregularity of the surface of the PSB. Grade 3: a large (>50% of CSA) area of hypoechogenicity with anechoic areas. Image to the right: large hypoechoic region with loss of long linear parallel echoes, with focal hyperechoic regions, and irregularity of the compact bone margin of the PSB.

Lesion severity was graded considering both echogenicity and CSA (Figure 4):

1. Grade 0 = normal: uniform normal echogenicity; long linear parallel echoes in longitudinal images.
2. Grade 1 = mild: less than 25% of the CSA of the ligament was hypoechoic with localised hypoechoic or anechoic lesions. Loss of linear echoes, reduced echogenicity in demarcated areas in longitudinal images.
3. Grade 2 = moderate: Hypoechoic or anechoic regions occupying 25–50% of the CSA of the ligament. Loss of long linear parallel echoes in longitudinal images and mild to moderate changes at the enthesis such as irregularity of the bone surface or hyperechoic regions in the SL.
4. Grade 3 = severe: Large anechoic and hypoechoic areas occupying >50% of the CSA of the ligament in transverse images. Hypoechoic or anechoic regions in longitudinal images and/or large hyperechoic regions (an avulsion or dystrophic mineralisation) in the SL and/or considerable irregularity of the bone surface at the enthesis.

It was also noted if hyperechoic foci were present and were or were not associated with acoustic shadowing.

#### 2.4.2. Radiological Interpretation

A diagnosis of osteoarthritis was based on the presence of periarticular osteophytes [26] without or with alterations in subchondral bone thickness or opacity, or joint space narrowing. The presence of entheseous new bone at the joint capsule attachments was also recorded.

Radiolucent areas in the PSBs, either focal or of linear shape, were classified as follows:

1. Linear radiolucencies originating from the abaxial surface (widened vascular channels [27]) subdivided into one linear radiolucent line or more than one linear radiolucent line.
2. Focal approximately oval or circular radiolucencies.

### 2.5. Follow-Up Ultrasonographic Examinations

Ultrasonography and power Doppler examinations were repeated every 45 days after injury. Suspensory branch lesions were classified as non-healing if there was no improvement in ultrasonographic appearance (for example, enlarged CSA, no reduction in lesion CSA, heterogeneous echogenicity and no improvement in lesion echogenicity or Doppler activity), or there was progressive deterioration, over three months of close follow-up, despite treatment and appropriate management.

### 2.6. Treatment and Follow-Up

#### 2.6.1. Modification of Exercise

Horses with grade 1 and 2 lesions were treated by decreasing those ridden exercises that increase fetlock extension, such as extended trot, or those that create asymmetric extension of a suspensory branch such as lateral exercises. Management was adapted to each horse and rider/trainer facilities and circumstances, as well as lesion severity and lesion progression, based on serial ultrasonographic examinations. In general, if total CSA, CSA of the lesion and power Doppler activity reduced, exercise was increased every two weeks. At three months, horses with mild lesions commonly returned to routine exercise, although minimising extended trot and lateral exercises. Horses with moderate lesions started trotting exercise after six weeks of walking exercise and were monitored closely clinically and ultrasonographically. If lesion CSA increased, exercise was reduced to walk. Horses with grade 3 lesions were initially restricted to hand walking exercise (30 min twice daily, in hand, on a treadmill or using an elliptical horse walker). Progress thereafter was individually tailored, dictated by clinical and ultrasonographic observations. For example, if the SL branch was enlarged, but there were no anechogenic regions or power Doppler signal, exercise was increased but close clinical and ultrasonographic monitoring continued. If the CSA of a branch was stable but there was a persistent anechogenic lesion, the exercise remained the same and the horse was reassessed after 15 or 30 days, depending on the owner’s choice. If there was no change after 15 or 30 days, the owner was advised that they could increase exercise with continued clinical and ultrasonographic monitoring, accepting the risk, but in the knowledge that any signs of not coping would result in a return to the previous exercise level.

When horses returned to moderate work, the work programmes were tailored to the level at which a horse was working and competing. For example, for an experienced horse competing at Intermediate level or above, it was advised that it should only perform extended gaits and lateral movements with which it was familiar in competition. For a less experienced younger horse, it was generally advised that extended paces and lateral movements should only be introduced when the SL branch was stable with no Doppler signal and the horse had been in progressive ridden work for at least one month. Riders were recommended to minimise repetitions and to have fitness days when horses performed a variety of exercises, each for two to three minutes, each using different muscle groups (for example, leg yield, walk–canter–walk transitions, raised trot poles). For the rehabilitation period, riders were advised to avoid deep or hard surfaces, to reduce the degree of sinking and impact, respectively. Horses were exercised after arena maintenance, before the surface was impacted by other horses, whenever possible. Some horses were exercised on an overground treadmill, but the use of a water treadmill was discouraged to avoid fetlock hyperextension. The use of a circular horse walker was also discouraged because lameness had been previously observed to deteriorate. For all horses, recommendations were made concerning trimming and shoeing, working directly together with a farrier, when possible, with particular focus on the correction of mediolateral and dorsopalmar(plantar) imbalance.

#### 2.6.2. Additional Treatments

With owner consent, three treatments using a radial pressure wave machine were applied every 15 days (2000 pulses per ligament, 11 MHz frequency and 3 BAR pressure) (Storz radial MasterPULSE, MP50 VET Dr.-Karl-Storz-Straße 34, 78532 Tuttlingen, Germany). Treatments were performed in a weightbearing position, focusing on the injured area, and varying the angle and moving the probe from proximal to distal over the affected regions. Sedation was not necessary in any horse, although there was a period of acclimation to noise and sensation prior to application.

Percutaneous electrolysis under ultrasonographic guidance was used in one horse because of a lack of improvement after radial pressure wave therapy. In horses in which a distal exostosis of a second or fourth metacarpal bone was interfering with the SL body and/or branch, surgical removal of the exostosis or the distal end of the bone was performed. In one horse with concurrent proximal suspensory desmopathy, osteostixis of the proximal aspect of the third metacarpal bone was also performed. One horse underwent tenoscopy of the digital flexor tendon sheath because of a concurrent lesion of the manica flexoria.

Horses with a concurrent diagnosis of symptomatic osteoarthritis of an MCP/MTP joint were treated by intra-articular medication:

- First-choice treatment was intra-articular administration of corticosteroids (triamcinolone, [6–12 mg/joint varying according to lesion severity and concomitant treatments], bethamethasone [6–18 mg/joint] or dexamethasone [6–18 mg/joint]) (variable manufacturers’ products for each corticosteroid were used). If more than one joint was treated, the dose was adjusted to a safe maximum dose [28].
- For more advanced osteoarthritis, polyacrylamide gel was administered (Arthramid Gel, Contura 301 Mallory Lane, Suite100, Fanklin, TN, USA).

Treatment was normally performed at the end of the course of radial pressure wave therapy, to avoid any interference with healing and to maximise pain relief at the beginning of exercise to minimise movement compensations.

#### 2.6.3. Management After Return to Full Work

It was recommended that horses should undergo clinical and ultrasonographic examinations, including power Doppler, every two months. The echogenicity, CSA of the SL branch and the CSA of any residual lesion were monitored. For those horses with osteoarthritis of an MCP or MTP joint, intra-articular medication was repeated if there were recurrent associated clinical signs (joint effusion, resentment of passive flexion, lameness or lameness induced by flexion).

#### 2.6.4. Acquisition of Follow-Up Data

Follow-up information was acquired by either re-examinations of the horses, followed by telephone contact with the owners and trainers over time, or by communication with the referring veterinarian. Competition records were also reviewed. Outcome was classified as 1. good if a horse returned to the pre-injury level of work or higher, 2. poor if a horse returned to a lower level of work compared with pre-injury or 3. retired from ridden work. The duration of follow-up ranged from four months (only horses with no response to treatment) up to five years. Data are presented relative to the first SL branch injury a horse sustained; a small proportion of horses made a complete recovery and subsequently sustained injury of another SL branch. The results for the second injury are presented separately.

### 2.7. Data Analysis

Relevant data were collated and stored in a Microsoft Excel spreadsheet (Office 365; Microsoft Corporation, Redmont, Washington, DC, USA). All statistical analyses were conducted using R Statistical Software (v4.4.2; R Foundation for Statistical Computing, Vienna, Austria. https://www.R-project.org accessed on 21 September 2025). The dataset was split into three subsets for analysis, depending on the primary observations of interest: (1) horse-level data that included information at the time of injury diagnosis; (2) limb-level data that included all limbs affected at the time of injury diagnosis and during subsequent follow-up and (3) branch-level data that included all branches affected at the time of injury diagnosis and during subsequent follow-up. Continuous variables (horse age, CSA, lesion CSA and lesion percentage) were checked for normality visually using histograms and formally using the Shapiro–Wilk test and were described using median with corresponding interquartile range (IQR) and range or mean ± standard deviation (SD), depending on distribution. The remainder of the variables were categorical and were described as proportions (%). An additional binary outcome variable was created representing good and bad outcomes, where poor and retired outcomes were combined into one category, to increase statistical power.

The proportion of forelimbs versus hindlimbs affected and the proportion of medial versus lateral branches affected were compared using the two-sample test for equality of proportions (without a continuity correction) based on the Z-test. The relationship between outcome and signalment, dressage level and clinical- and lameness-related variables was assessed using Pearson’s Chi-squared (*Χ*^2^) test (with Yate’s continuity correction) or the Fisher’s exact test (when any one observed frequency in the contingency table ≤5). Where an initial significant overall association was found for categorical variables with more than two levels (*Χ*^2^ or Fisher’s exact test *p* ≤ 0.05), pairwise comparisons were carried out post hoc to identify exactly which levels were different (including Bonferroni adjustment for multiple comparisons). The same methods were used to explore relationships between clinical- and lameness-related variables. The relationship between continuous and categorical variables (e.g., horse age and outcome) was assessed using the Kruskal–Wallis rank sum test. Where an initial significant overall association was found for categorical variables with more than two levels (Kruskal–Wallis *p* ≤ 0.05), this was followed up with a post hoc Dunn’s test with Bonferroni adjustment for multiple comparisons.

A significance level of *p* ≤ 0.05 was used without adjustment for multiple comparisons [29]. Even though some of the observations were obtained from different limbs of the same horse, for the purposes of this analysis, all observations were treated as independent.

## 3. Results

### 3.1. Demographic Data and Clinical Examination

A total of 70 horses were included in the study, 43 Warmbloods (61.4%), 22 Iberian (31.4%) and 5 cross breeds (7.1%). There were 45 geldings (64.3%), 22 stallions (31.4%) and 3 mares (4.3%). There were 29 young or amateur horses (41.4%), 21 horses at Prix St Georges level (30%), 10 horses at Intermediate I or II or U25 (14.3%) and 10 horses at Grand Prix level (14.3%). Median age was 9 years (IQR 7,12; range 3,17). None of the amateur or U25 horses were ‘schoolmasters’ that had been downgraded from a higher level of training/competition.

There were 89 branches affected, and 74 limbs. Sixty-one horses had one branch affected, five horses had both medial and lateral branch injuries in the same limb, one horse had both branches affected in each forelimb and three horses had several branches affected in different limbs. The left forelimb was affected in 23 limbs (31.3%), the right forelimb in 21 (28.4%), the left hindlimb in 13 (17.6%) and the right hindlimb in 17 (24.3%). There was a higher prevalence of lateral branch injuries, 67/89 (75.3%), compared with medial branches, 24/89 (27%) (*p* < 0.001). The prevalence of injuries in forelimbs (44/74, 59.5%) and hindlimbs (31/74, 41.9%) was significantly different (*p* = 0.01).

A total of 40 out of 89 branches (44.9%) had associated acute inflammatory signs such as localised heat and oedema. A total of 17 out of 89 injured branches (19.1%) were not palpably enlarged; 27/89 branches (30.3%) were mildly enlarged and 45/89 (50.6%) were severely enlarged. Distension of the MCP/MTP joint capsule was detected in 45/74 (60.8%) of affected limbs, and distension of the digital flexor tendon sheath in 15/74 (20.3%) limbs. A painful response was elicited by firm pressure applied to the affected branch for 75/89 (84.3%) branches. Passive flexion of the distal aspect of the limb was resented in 49/74 limbs (66.2%). One horse had hyperextension of the affected fetlock.

Seven horses were not evaluated at trot because of clinical signs indicative of acute SL branch desmitis. Seven horses did not exhibit lameness. The median lameness grade for affected limbs of horses evaluated dynamically was 2/5 (range 0, 3). A total of 5 out of 31 (16.1%) affected hindlimbs were abducted as protracted. A positive response to a distal limb flexion test was observed in 40/74 (54.1%) affected limbs. A total of 5 limbs out of 74 (6.8%) were positive to flexion with no associated osteoarthritis of an MCP/MTP joint.

### 3.2. Diagnostic Ultrasonography

The lesion was localised distally in 59/89 branches (66.3%), in the mid region in 14/89 branches (15.7%) and proximally in 4/89 (4.5%). There were lesions in both the proximal and mid areas in one branch (1.1%), mid and distal lesions in six branches (6.7%) and all areas were affected in five branches (5.6%). The abaxial area was involved in 28/89 branches (31.5%), the axial area in 25/89 branches (28.1%.), the central area in 9/89 (10.1%) and the dorsal region in 5/89 branches (5.6%). In 21/89 branches (23.6%), lesions were diffuse. Periligamentous fibrosis was present around six of 89 branches (6.7%).

In total, 20/89 branch injuries (22.5%) were classified as mild (grade 1), 43 moderate (grade 2) (48.3%) and 26 severe (grade 3) (29.2%). There were 13/89 branches (14.6%) with hyperechoic regions causing acoustic shadowing, while 15 (16.9%) had hyperechoic regions without acoustic shadowing. The lesion was mainly anechogenic in 22/89 branches (24.7%), hypoechoic in 31/89 branches (34.8%) and had mixed echogenicity in 36/89 branches (40.4%).

The mean CSA of affected branches was 1.99 cm^2^ (SD ± 0.07). The mean lesion CSA was 0.96 cm^2^ (SD ± 0.03). The lesion CSA as a percentage of the SL CSA had a mean of 21.0% (SD ± 0.96).

### 3.3. Power Doppler Examination

**Figure 5:**
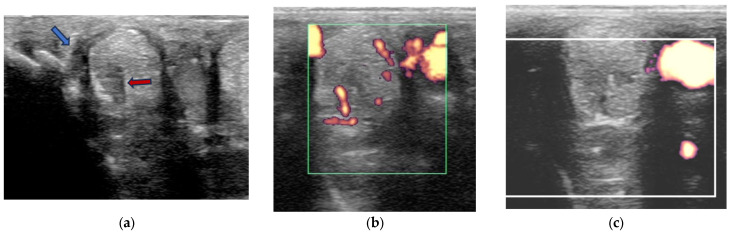
Twelve-year-old Warmblood mare; (**a**) B-mode transverse ultrasonographic image of the left forelimb lateral suspensory branch five days after injury. There is a well-defined hypoechoic region (red arrow) within the branch, and a hypoechoic region subcutaneously consistent with periligamentous oedema (blue arrow); (**b**) power Doppler image at the same level as (**a**). There is severe power Doppler signal. (**c**) Transverse power Doppler image one month after injury; there is no periligamentous oedema, the lesion in the branch is less well-defined and there is no power Doppler signal within the branch.

**Figure 6:**
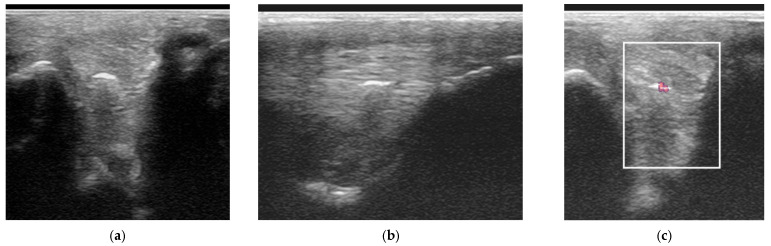
Twelve-year-old Warmblood mare with right hindlimb lameness and abduction of the limb during protraction. (**a**) Transverse, (**b**) longitudinal (distal to the right) and (**c**) transverse power Doppler images of the lateral branch of the suspensory ligament. There is a linear hyperechoic region causing acoustic shadowing. There is mild power Doppler signal localised around the hyperechoic region.

**Figure 7:**
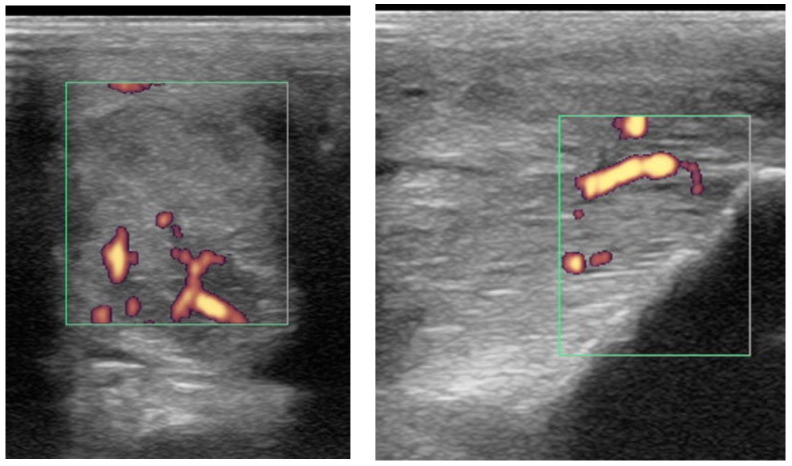
Transverse (**left**) and longitudinal (**right**) ultrasonographic images of the lateral branch of the suspensory ligament (SL) of a hindlimb of a 15-year-old purebred Spanish horse (PRE), acquired 2 weeks after injury, with a non-healing suspensory branch desmitis. The SL branch is enlarged with large hypoechoic regions and loss of long linear parallel echoes in the longitudinal image. There is also substantial subcutaneous echogenic tissue consistent with periligamentous fibrosis. Power Doppler signal was graded severe and persisted despite treatment. The horse was retired.

**Figure 8:**
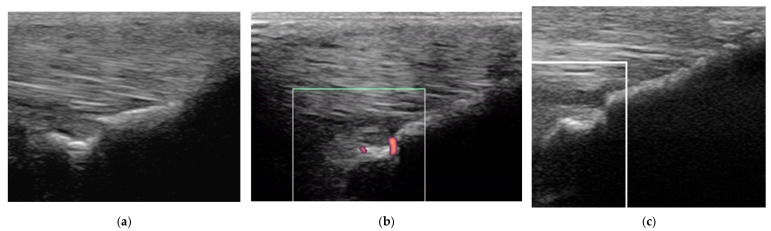
Longitudinal ultrasonographic images of the lateral branch of the suspensory ligament (SL) of a right forelimb of a four-year-old gelding (**a**,**b**) before treatment and (**c**) six months later; proximal is to the left. (**a**) There is loss of long linear parallel echoes in the dorsal aspect of the SL close to the enthesis. There is irregularity of the surface of the proximal sesamoid bone (PSB). (**b**) There was mild Doppler signal at the enthesis on the apex of the PSB. (**c**) At six months after treatment, a hypoechoic lesion persists, but there is no longer any power Doppler signal.

Power Doppler examination was performed in 62/89 branches (67.4%). Doppler signal was present in 59/62 (95.2%) branches (Figure 5, Figure 6, Figure 7 and Figure 8) and absent in three branches. At the initial examination, Doppler signal was classified as mild in 22/62 branches (35.5%), moderate in 25/62 (40.3%) and severe in 12/62 (19.4%) branches. Doppler signals persisted in follow-up examinations, despite treatment, in 17/62 branches (27.4%) (Figure 7).

### 3.4. Radiographic Examination

Sixty-two limbs were examined radiographically. One linear radiolucent line was observed in the PSB ipsilateral to the injured SL branch in 32/62 (50.8%) limbs and more than one radiolucent line was detected in 22/62 (34.9%) PSBs. A well-defined circular or oval radiolucent area was seen in 19/62 (30.2%) PSBs and more than one focal lesion in 19/62 (30.2%). A second or fourth metacarpal or metatarsal exostosis was identified in three limbs, mineralisation proximal to the apex of the PSB in one limb and entheseous new bone on the PSB in one limb.

### 3.5. Other Concurrent Injuries in the Limb with a Branch Injury

Branch desmitis was considered primary in 25/74 limbs (33.8%) and associated with pre-existing symptomatic osteoarthritis of the MCP/MTP joint in 49/74 limbs (66.2%). Other concomitant lesions were digital flexor tendon sheath tenosynovitis in 5/74 limbs (6.8%), collateral desmitis of the fetlock joint in 7/74 limbs (9.5%), exostosis of the second or fourth metacarpal/metatarsal bones in 5/74 limbs (6.8%) and desmitis of the origin of the SL in 8/74 limbs (10.8%).

### 3.6. Treatment and Outcome

Sixteen horses were treated conservatively by rest and hand walking exercise or restricted paddock turnout. Radial pressure wave therapy was performed in 56 horses either alone or combined with intra-articular medication of the ipsilateral MCP or MTP joint in nine. Surgical removal of an exostosis on the second or fourth metacarpal or metatarsal bone was performed in three horses, followed by radial pressure wave therapy in two. The total exceeds 70 because of combined treatments.

Follow-up outcome was good in 44/70 horses (62.9%), poor in 13/70 (18.6.%) and 13/70 horses (19.1%) were retired due to no response to treatment. Age (*p* = 0.85), breed (*p* = 0.24), training level (*p* = 0.53), number of injured branches (*p* = 0.27) or number of limbs affected (*p* = 0.62) were not associated with the follow-up outcome (Table 1).

**Figure 9:**
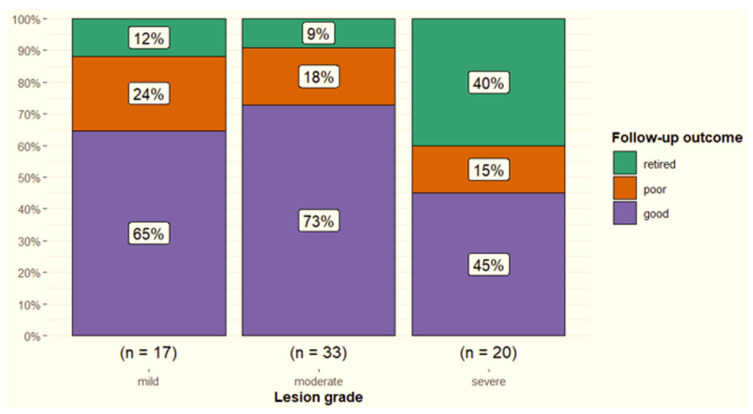
Follow-up outcomes for 70 dressage horses with desmitis of one or more suspensory ligament branches, based upon the worst ultrasonographic grade (grade 1 = mild, grade 2 = moderate, grade 3 = severe) assigned. Good = a horse returned to the pre-injury level of work or higher; poor = a horse returned to a lower level of work compared with pre-injury; retired = retired from ridden work because of the injury.

Localisation of the lesion in a transverse image was not associated with outcome (*p* = 0.65), although 27% of horses with diffuse injuries were retired compared with 12% of those with dorsal injuries and none of those with abaxial injuries. Lesion grade was not associated with outcome (*p* = 0.07). However, 40% of 20 horses with severe lesions were retired, compared with 9% of 33 horses with moderate lesions and 12% of 17 horses with mild lesions (Figure 9).

The CSA of the affected branch (*p* = 0.96), the CSA of the lesion (*p* = 0.28) and the lesion CSA as a percentage of the SL CSA (*p* = 0.40) were not associated with follow-up outcome. The presence of acoustic shadowing was not associated with outcome (*p* = 0.16). The presence of periligamentous fibrosis did not affect outcome (*p* = 0.60); however, 33% of the horses with periligamentous fibrosis were retired compared with 17% of horses without periligamentous fibrosis.

**Figure 10:**
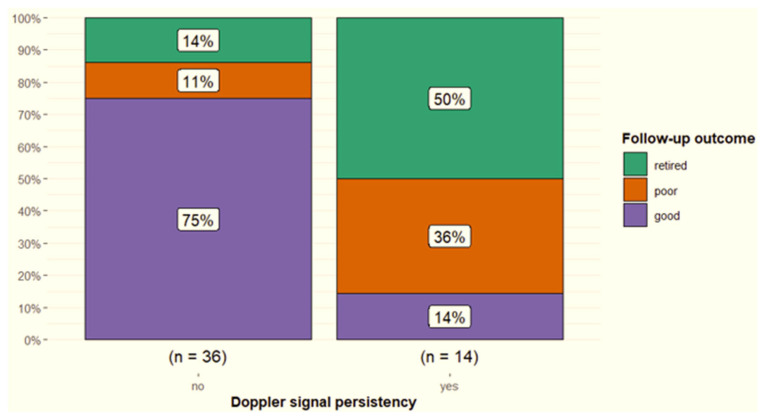
Follow-up outcome for horses (n = 50) with suspensory ligament branch injuries that underwent power Doppler examination comparing those in which signal disappeared at 45 days after the initial examination with horses in which signal persisted. Good = a horse returned to the pre-injury level of work or higher; poor = a horse returned to a lower level of work compared with pre-injury; retired = retired from ridden work because of the injury.

The severity of power Doppler signal was not associated with outcome (*p* = 0.20); however, 56% of horses with severe power Doppler signal were retired compared with 22% with moderate signal and 12% with mild signal. Persistent Doppler signal over time was associated with follow-up outcome (*p* < 0.001) (Figure 10). In particular, post hoc testing showed that significant differences were present for associations between the good (*p* = 0.001) and retired (*p* = 0.04) outcome categories and Doppler signal persistency, but not the poor (*p* = 0.25) outcome category. Only 6/17 (35.2%) of horses with persistent Doppler signal (Figure 7) had a good outcome, whereas 11/17 (64.7%) had a poor outcome or were retired.

The presence of co-existent osteoarthritis of an MCP/MTP joint did not affect the follow-up outcome (*p* = 0.73), nor did radiological abnormalities of the PSBs (*p* = 0.69).

**Figure 11:**
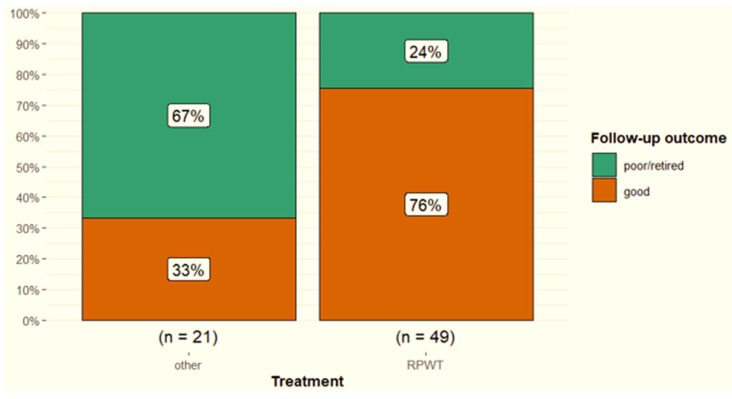
Follow-up outcomes for 70 dressage horses with desmitis of one or more suspensory ligament branches, comparing the outcome for those horses receiving a course of radial pressure wave therapy (RPWT) with other treatments. Good = a horse returned to the pre-injury level of work or higher; poor = a horse returned to a lower level of work compared with pre-injury; retired = retired from ridden work because of the injury.

Conservative treatment was elected by owners in 16 horses (21 branches) of which 5/16 (31.3%) had a good outcome, 6/16 (37.5%) had a poor outcome and 5/16 were retired (31.3%). In total, 42 horses (54 branches) received radial pressure wave therapy of which 32/42 (76.2%) horses had a good outcome, 5/42 (11.9%) had a poor outcome and 5/42 (11.9%) were retired. Horses treated with radial pressure wave therapy and monitoring had a better outcome compared with those receiving other treatments (*p* = 0.003) (Figure 11). Three horses that had surgical treatment had a good outcome, one poor and one was retired.

### 3.7. Recurrent Injuries

**Figure 12:**
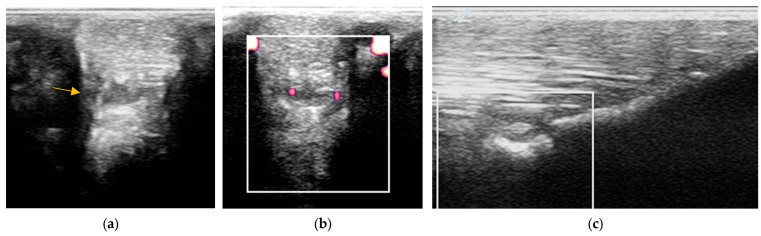
The same suspensory ligament branch as Figure 8, acquired four years after the initial injury. (**a**) Transverse image at the mid region, (**b**) transverse power Doppler image at the same level as (**a**,**c**) longitudinal power Doppler image of the distal region (proximal is to the left). (**a**) There is a new injury at a more proximal site (arrow) than the original injury, with mild Doppler activity (**b**). At the original injury site (**c**), a hypoechoic lesion is still apparent, but no Doppler signal was detected. There was localised swelling at the injury site, and distension of the metacarpophalangeal joint capsule.

Two horses had recurrent desmitis of the same injured branch as the original injury, having returned to full work, one and four years after the initial injury, respectively (Figure 12). Both horses had a good outcome.

### 3.8. Second Injuries

Six horses that had a good outcome from their initial injury subsequently sustained an injury in a different SL branch between six months and four years later (Table 2). Of these six horses, four had a good outcome and two were retired.

## 4. Discussion

### 4.1. Results Related to Hypotheses

In accordance with the hypothesis, the prevalence of lateral branch injuries was higher than medial branches. Distal lesions and involvement of the insertion onto the PSBs were more common than proximal injuries. Contrary to the hypothesis, ultrasonographic grade was not significantly associated with follow-up outcome, although horses with severe injuries had a worse outcome than horses with mild or moderate injuries. Contrary to the hypothesis, horses with abaxial lesions did not have a better outcome than horses with other anatomical sites of lesions.

#### 4.1.1. Lateral Versus Medial Branches and Forelimbs Versus Hindlimbs

In a previous study of a mixed population of sports horses, there was no difference in prevalence between medial and lateral branch injuries [1]. Likewise, in a study of asymptomatic western performance Quarterhorses, there was a similar prevalence of medial and lateral branch lesions [30]. However, in a United States of America (USA) study of Thoroughbred flat racehorses, medial branch desmitis was more prevalent than lateral [9]. In asymptomatic elite showjumpers, there was a higher prevalence of medial branch injuries graded 2 or 3 (0–3 scale) compared with lateral branch injuries [12] and, as suggested by the authors, the prevalence may be discipline-related.

Lateral exercises such as shoulder in and half-pass are a common part of any training session in dressage beyond basic levels but are uncommon in other equestrian sports disciplines. During lateral movements in the propulsion phase of the outside limb, the lateral branch of the SL is under more tensile force than the medial branch as the limb propels the horse towards the opposite side [6], which may explain the predominance of lateral branch injuries in the current study.

In the current study, SL branch injuries in forelimbs (61%) were more prevalent than in hindlimbs (41%). In a study of asymptomatic elite showjumpers, SL branch injuries were more prevalent in hindlimbs compared with forelimbs [12], whereas in asymptomatic western performance Quarterhorses, forelimb injuries were more prevalent than in hindlimbs [30]. In a clinical study of SL branch injuries, there was a similar proportion of forelimb and hindlimb injuries [1]; however, in contrast to the current study, forelimb injuries were overrepresented in event horses (76%) and hindlimb injuries in dressage horses (87%). These differences might be explained by studies performed in different countries, with different populations of horses trained on varied surfaces, and perhaps hindlimb lameness being overlooked by riders and trainers in the catchment region of the current study.

#### 4.1.2. Distal Lesions: The Enthesis

Distal lesions were the most common location of injury in the current study. Previous reports of subclinical injuries in flat racehorses [10], National Hunt racehorses [11] and showjumpers [12] did not specify the proximodistal location of injuries but all figures illustrated lesions close to the insertion on the PSB, so it is reasonable to assume that this was the most common site of injury. In a clinical study of 71 sports horses, most injuries involved the distal third of the ligament, but the proximodistal extent of lesions varied considerably [1]. The enthesis is one of the most studied areas in human orthopaedics due to the high prevalence of injuries at this location related to the different tissue types and properties of the tissues at the bone–ligament interface, with associated stress concentration [3,4,31]. Likewise, the proximal enthesis of the suspensory ligament in equine hindlimbs is a four-layered structure consisting of ligament, uncalcified fibrocartilage, calcified fibrocartilage and bone [32], probably reflecting a stress dissipation mechanism. The infrastructure of the enthesis on each PSB has not been studied. Hyperextension of the MCP and MTP joints occurs during many of the exercises trained and repeated in dressage, subjecting the branches to high tensile loads repetitively [6]. In addition, 11% of affected limbs in the current study had concurrent proximal suspensory desmopathy that could affect the elastic properties at this level, translating compensatory forces distally to the branches.

#### 4.1.3. Ultrasonographic Parameters and Outcome

Grade 2 (moderate) ultrasonographic lesions were the most common injuries in the current study. In previous surveys of flat racehorses [10], National Hunt racehorses [11] and showjumpers [12] using similar grading schemes, grade 2 lesions that were subclinical have been identified. However, for inclusion in the current study, SL branch injuries had to be a known source of pain contributing to lameness.

The grading system is easy to use and is repeatable; therefore, monitoring injuries over time can help to give a prognosis. Horses with grade 3 ultrasonographic lesions had a worse follow-up outcome than those with grade 1 or 2 injuries. This outcome was not statistically significant (*p* = 0.07) but may be of biological significance. Although it is reasonable to assume that severe injuries would be more difficult to treat, and the outcome would be worse compared with milder injuries, some acute severe injuries do heal with a functional tissue, with 45% of horses with a grade 3 branch lesion in the current study having a good follow-up outcome.

Hyperechoic areas without acoustic shadowing (17% of branches), representing fibrosis, and with acoustic shadowing (15% of branches), representing dystrophic mineralisation, were observed with similar frequency in the current study. Although focal mineralisation was illustrated in an SL branch of an asymptomatic showjumper [12] and in an SL of an asymptomatic National Hunt racehorse [11], the frequency of occurrence of fibrosis or mineralisation was not documented, which may suggest that these were uncommon observations. In a USA-based study of Thoroughbred racehorses at major sales, focal mineralisation was seen in at least one SL branch of 2% and 8% of yearlings and 2-year-old horses, respectively, whereas focal hyperechoic lesions without acoustic shadowing were identified in 18% of yearlings and 24% of 2-year-olds [9,33]. Hyperechoic foci without acoustic shadowing did not influence outcome, whereas mineralisation was associated with lower total earnings and earnings per start compared with unaffected horses [9]. In a clinical study of sports horses, hyperechoic foci without acoustic shadowing were identified in only 4% of the affected branches and mineralisation with acoustic shadowing was detected in only 2% of the injured branches [1]. The relatively high frequency (15%) of focal mineralisation in the current study may reflect innate differences in the horse populations. In human medicine, for example, with rotator cuff injuries, it is recommended that power Doppler should be used to assess if there is any vascular activity surrounding areas of fibrosis or dystrophic mineralisation that could represent inflammation [34]. Association between pain and power Doppler activity has been reported for rotator cuff injuries [34], but this may not be the same for all soft tissue injuries [35].

### 4.2. Power Doppler

Doppler and power Doppler are used to detect neovascularisation [36,37,38,39]. Power Doppler detects smaller vessels than conventional Doppler and is dependent on the number of cells that cross but is not influenced by the direction of the flow [36]. The presence of neovascularisation is normal after injury and has been correlated with the presence of neurovascular vessels, although the exact association with pain is still subject to debate and is structure-dependent [38,39,40,41,42,43]. After injury, an intraligamentous haematoma may develop, followed by neovascularisation. Doppler activity is normally intense for weeks after injury [43,44]; however, if the healing process is appropriate, these vessels disappear gradually over time [44].

The use of power Doppler in human sports medicine has been widely reported [35,36,37,39,40]. In contrast, the use of power Doppler in equine sports medicine has not been well studied. The suspensory branches of 10 lame limbs and 24 non-lame limbs of eight lame horses and five non-lame horses were examined using B-mode ultrasonography and with power Doppler, with the limbs non-weightbearing [13]. The SL branches that appeared normal using B-mode ultrasonography had no Doppler signal, whereas structurally abnormal branches in both non-lame and lame limbs had power Doppler signal [27]. In humans, the presence of Doppler signal in tendons of asymptomatic elite athletes after intense effort has been documented [35]. It was suggested that this may represent remodelling of the tendon [35]. One hundred and sixty-eight SL branches of 21 non-lame western performance Quarterhorses were assessed using both B-mode ultrasonography and power Doppler [30]. Using B-mode ultrasonography, lesions were identified in 25 forelimb and 10 hindlimb SL branches. Structurally normal SL branches had no power Doppler signal, whereas power Doppler signal was detected in 22 of 35 SL branches with lesions (63%).

In the current study, power Doppler examinations were performed with the limbs bearing weight because the signal was clearly visible in this position and it was easier to detect hypoechoic regions occupied by vessels between fibres. If power Doppler signal was not present in images acquired in a weightbearing position, then the examination was repeated with the limb in flexion.

In the current study, based on the power Doppler examinations, most of the purely anechogenic linear areas between more echogenic tissue were occupied with blood vessels in acute injuries or in non-healing lesions. In contrast, large anechogenic areas were not commonly invaded by vessels. It may be important to consider that small hypoechoic round or linear areas seen commonly during ultrasonographic examinations may represent vessels in the proximity of lesions rather than interfibrillar oedema or ruptured fibres [35].

### 4.3. Monitoring: The Use of Power Doppler in Non-Healed Cases; Uncontrolled Inflammatory Response

In the current study, most SL branches with persistent hypoechoic lesions over time, which suggested an absence of healing, also maintained severe Doppler signal affecting a large part of the ligament. No decrease in signal intensity and area affected was observed during serial examinations. In human medicine, prolonged vascularity is common in persistent inflammatory enthesitis, such as in rheumatoid patients [39]. It is thought that this reflects a dysregulation between inflammatory mediators and inhibitors, instead of a normal process of repair and remodelling after injury. The tendon or ligament remains in a persistent inflammatory status with pain on palpation [3].

In the current study, the non-healed SL branches occurred more commonly in hindlimbs than in forelimbs (n = 4 and n = 2, respectively). In the hindlimbs, all non-healed SL branches had an increased size, periligamentous fibrosis, severe heterogenicity and persistent severe power Doppler signal despite treatment. Periligamentous fibrosis increased over time, probably in an attempt to limit fetlock hyperextension. The non-healing forelimb SL branches remained enlarged with heterogenous echogenicity and persistent power Doppler signal, as reported in human in degenerative lesions [35].

### 4.4. Periligamentous Fibrosis

Overall, in the current study, the prevalence of periligamentous fibrosis was higher in hindlimbs than in forelimbs, as has previously been reported in both asymptomatic showjumpers [12] and lame sports horses [1,13]. In contrast to a previous study [1], the presence of periligamentous fibrosis in the current study did not significantly influence outcome, but this may reflect the low prevalence of periligamentous fibrosis. Periligamentous fibrosis may still be of potential biological importance with respect to prognosis and was observed to increase in association with non-healing lesions.

### 4.5. Primary Fetlock Osteoarthritis, Secondary Branch Desmitis; Compensatory Overload?

A relationship between suspensory branch desmitis and fetlock region pain, particularly related to osteoarthritis, has been previously reported [1,45]. In the current study, fetlock osteoarthritis often existed when suspensory branch desmitis was first recognised. In a postmortem study of 73 right MCP joints from horses aged 0.4–23 years, the proximal articular surface of the proximal phalanx was quantified using a cartilage degeneration index [46]. The cartilage degeneration index increased from lateral to medial and from central to dorsal. It was suggested that loading of the proximal phalanx is higher medially than laterally because of the excentric position of the centre of gravity of the horse in relation to the limb. The combination of biomechanical loading superimposed on age-related degeneration may explain the distribution of osteoarthritis. Predilection for injury of the cartilage and/or subchondral bone of the medial fovea of the proximal phalanx and the medial condyle of the third metacarpal bone in sports horses has been reported in numerous clinical studies [47,48,49,50]. Pain associated with osteoarthritis or subchondral bone injury of an MCP or MTP joint could result in altered loading of the joint predisposing to SL branch injury.

In association with SL branch injuries, there is often effusion in the MCP or MTP joints [1], involving particularly the palmar or plantar recess. Chronic effusion and release of inflammatory mediators may contribute to the initiation or progression of osteoarthritis. Postmortem examination of 24 limbs with chronic SL branch injuries, but without radiological evidence of osteoarthritis, revealed hyperplasia of the synovium and chronic adhesions in the palmar/plantar recess of the MCP or MTP joints of variable severity in all limbs [51]. Chronic wear lines in the condyles of the third metacarpal or metatarsal bone were widespread.

### 4.6. Treatment, Rehabilitation and Outcome of SL Branch Desmitis

In the current study, 62.9% of horses had a good outcome following either conservative management or treatment with radial pressure wave therapy, combined with intra-articular medication of the fetlock when indicated. In a referral population of mature sports horses, 43.5% of horses with suspensory branch desmitis and no concurrent injuries had a good outcome following a variety of treatments combined with rest and controlled exercise, with sustained return to competition for at least one year and up to five years at the time of acquisition of follow-up information [1]. Sixty-nine Thoroughbred flat racehorses between 1 and 6 years of age (median 2 years) with suspensory branch desmitis were treated with intralesional allogeneic mesenchymal stem cells followed by three or four treatments with bone marrow-derived mesenchymal stem cells [52]. There was a high success rate in these young athletes with 71% (95% confidence intervals: 59–81%) racing after injury with a mean career length of 30 months. In an experimental model of SL branch injuries [53] performed in 12 mature Warmbloods, differences were observed in the healing of lesions treated with high-power laser compared with control lesions in a short-term (six months) follow-up study; however, the mechanical properties of the tissue were not assessed. The outcome of treatment of mature sports horses with laser therapy has been reported for a variety of tendon and ligament injuries [54], but the outcome for those with SL branch desmitis was not specified and additional therapies other than laser were also used. A mixed population of 18 horses with dorsal intra-articular lesions of an SL branch were treated by arthroscopic debridement with 72% returning to former function, but the level of work was not specified [2]. Surgical debridement of longitudinal palmar abaxial lesions was performed in 29 Thoroughbred flat racehorses 1 to 8 years of age (mean 2.4 years) and 68% returned to racing [55].

Direct comparisons among studies are difficult given the differences in age, work disciplines, level of work, lesion type and severity and the presence or absence of other injuries that may have an important influence on outcome, such as pre-existing osteoarthritis. However, the superior results of the current study involving first-opinion clinical practice compared with the referral sports horse population of Marneris [1] may reflect earlier recognition of less severe injuries. In the current study, only one horse had hyperextension of an MCP or MTP joint at the time of injury diagnosis, whereas in the study of Marneris et al. [1], 22% of horses had hyperextension of one or both MTP joints, reflecting major SL dysfunction. This highlights the potential importance of regular monitoring of sports horses, as previously documented [21].

The role of radial pressure wave treatment in the outcome of the horses in the current retrospective study is difficult to determine because treatment modalities were influenced by owner choice. Use of radial pressure wave or extracorporeal shockwave therapy is widespread, but the quality of documented clinical studies is limited and largely restricted to proximal suspensory desmitis and not SL branch injuries [56,57,58,59,60]. There are no randomised blinded controlled clinical trials. Of the horses in the current study that had SL injuries that failed to heal, none responded to radial pressure wave therapy. If there is dysregulation of inflammatory mediators, stimulation of cells by radial pressure wave therapy may not be as effective as in more typical damaged tissue.

### 4.7. Is Prevention of Injury Possible?

Suspensory branch desmitis is a potential high-risk injury for dressage horses. There are a number of factors that may have the potential to reduce the risk of injury, including the maintenance of good foot balance [19,61], avoiding muscle fatigue [62] and management of pain, which may result in overload of the SL branches (for example, pain associated with osteoarthritis of an MCP or MTP joint).

Regular comprehensive routine examinations of the musculoskeletal system may facilitate early detection of mild injuries [21]. Subclinical abnormalities may precede an active episode of desmitis; therefore, routine ultrasonographic examinations combined with power Doppler ultrasonography could be scheduled over the training and competition seasons for the early detection of potentially significant lesions, with appropriate modifications of the training programme. Recognition and recording of the subclinical ultrasonographic findings of each athlete would help to monitor any further changes over time. Appropriate maintenance of arena surfaces [20,63], avoiding over-training, utilising cross training [19,20] and limiting the amount of work in extended paces [17,21] may all be beneficial. Training and competition schedules need to factor in adequate recovery time. Progressive development of appropriate muscle strength and coordination to provide stability and avoidance of over-training are crucial.

Exercises to improve muscle function have been effective for injury prevention in human athletes [64]. Exercises to improve proximal muscle support and impact absorption, particularly during lateral exercises, may help to decrease the prevalence of SL branch injuries in horses. Such exercises include walking up and down and across gradients, raised pole exercises at walk (not only in straight lines but also on bending lines) and trot and water treadmill exercise at an appropriate water depth tailored to each individual’s gait characteristics. There should be particular care when introducing new exercises, especially lateral exercises, to avoid excessive repetitions.

### 4.8. Limitations of the Study

As with any retrospective clinical study, there were limitations. The sample size was small, which limited the statistical power and prevented the use of multivariable analysis for factors such as outcome. A single clinician performed the clinical assessments and there may have been biases in decision-making, but it ensured a consistent clinical approach and recording of observations. Intra-articular and intrathecal anaesthesia were not routinely performed due to time constraints and working in a ‘field’ environment. The limbs were not clipped for ultrasonographic examinations, and a stand-off pad was not used, which may have limited image quality, especially in the near field. Not all horses underwent power Doppler examination. There was the potential for owners’ bias in the selection of treatments and determining whether a horse could resume full training. Owner compliance with treatment and management recommendations could not be guaranteed. The quality of trimming and shoeing, training and management techniques and arena surface management were variable. Other concurrent injuries may have contributed to the clinical outcome. The duration of follow-up was variable and shorter than a previous study of SL branch injuries [1] and survival analysis was not performed.

## 5. Conclusions

Suspensory ligament branch injuries are potentially career-limiting injuries in dressage horses and may occur as primary injuries or develop after pre-existing osteoarthritis of an MCP or MTP joint. Most ultrasonographic parameters of SL branch injuries were not associated with functional outcome, although the presence of periligamentous fibrosis resulted in a smaller percentage of horses having a good outcome compared with horses without periligamentous fibrosis. Power Doppler examination is a potentially useful tool for monitoring the response to treatment, with long-term persistence of power Doppler signal associated with a reduced likelihood of a good outcome. In this study, 63% of horses were able to return to full function at the same level as pre-injury or higher. This favourable result may reflect individually tailored rehabilitation programmes combined with regular clinical and ultrasonographic monitoring. Although horses that received radial pressure wave therapy had a superior outcome than other treatments, a randomised controlled clinical trial is required to verify whether radial pressure wave therapy influences the outcome.

## Figures and Tables

**Table 1:** Factors potentially affecting outcome of 70 dressage horses with suspensory ligament (SL) branch injuries. Significant *p* values are highlighted in bold. CSA = cross-sectional area; MCP = metacarpophalangeal; MTP = metatarsophalangeal; PSB = proximal sesamoid bone.

Factors Potentially Associated with Outcome		*p*-Value
Horse factors	Age	0.85
	Breed	0.24
	Training level	0.53
	Number of injured branches	0.27
	Number of limbs affected	0.62
Ultrasonography	Localisation of lesion	0.65
	Lesion grade	0.07
	CSA of injured branch	0.96
	CSA of lesion	0.28
	CSA of lesion as a percentage of SL CSA	0.40
	Acoustic shadowing	0.16
	Periligamentous fibrosis	0.60
	Severity of power Doppler signal	0.20
	Persistence of power Doppler signal over time	**<0.001**
Radiography	Osteoarthritis of MCP/MTP joint	0.73
	Radiological abnormalities of PSBs	0.69
Treatment	Radial pressure wave treatment versus other	**0.003**

**Table 2:** Summary data of the outcomes for six horses that had a good outcome from their initial injury but subsequently sustained an injury in a different suspensory ligament branch between six months and four years later. LF = left forelimb; RF = right forelimb; LH = left hindlimb; RH = right hindlimb; PSD = proximal suspensory desmitis. Good outcome = return to full work at same level as pre-injury or higher. Injury grade 1 = mild; 2 = moderate; 3 = severe.

Horse	Initial Injury	Injury Grade	Outcome	Second Injury	Outcome	Third Injury	Outcome
2	LH Lateral	3	Good	RH Lateral	Good		
4	LF Medial	2	Good	RH Lateral	Good	Recurrent RH Lateral	Good
6	RF Lateral	3	Good	LH Lateral	Retired		
43	LH Medial	3	Good	RF Lateral	Good		
47	LF Lateral	3	Good	LH Lateral	Good		
58	LF Lateral	2	Good	RF Lateral and PSD	Retired		

## Data Availability

Anonymised data are available from the authors upon reasonable request.
